# Ultrasensitive and Regenerative Transistor Sensor Based on Dynamic Covalent Chemistry

**DOI:** 10.3390/s22186947

**Published:** 2022-09-14

**Authors:** Ban-Peng Cao, Changhao Dai, Xuejun Wang, Qiang Xiao, Dacheng Wei

**Affiliations:** 1 Jiangxi Key Laboratory of Organic Chemistry, Jiangxi Science and Technology Normal University, Nanchang 330013, China; 2 State Key Laboratory of Molecular Engineering of Polymers, Department of Macromolecular Science, Fudan University, Shanghai 200433, China

**Keywords:** ultrasensitive, regenerative, dynamic covalent chemistry, field-effect transistor, selective

## Abstract

Field-effect transistor (FET) sensors require not only high sensitivity but also excellent regeneration ability before widespread applications are possible. Although some regenerative FETs have been reported, their lowest limit of detection (LoD) barely achieves 10^−^^15^ mol L^−^^1^. Here, we develop a graphene FET with a regenerative sensing interface based on dynamic covalent chemistry (DCvC). The LoD down to 5.0 × 10^−^^20^ mol L^−^^1^ remains even after 10 regenerative cycles, around 4–5 orders of magnitude lower than existing transistor sensors. Owing to its ultra-sensitivity, regeneration ability, and advantages such as simplicity, low cost, label-free and real-time response, the FET sensor based on DCvC is valuable in applications such as medical diagnosis, environment monitoring, etc.

## 1. Introduction

Field-effect transistors (FETs) have been developed to satisfy the requirements of modern electronics technology [1,2,3]. Due to their ability to provide low-cost, high sensitivity, real-time, and label-free detection of an analyte, FETs present bright prospects for widespread applications in physical [4], chemical [5], and biological sensing fields [6]. The sensing principle of FETs is rooted in the changes of potential and conductivity in the channel, which are related to physical and/or chemical interactions between probes and analytes at the interface [7,8,9,10]. To enhance the sensitivity, it is required to reinforce the interaction by utilizing a partially neutralized probe [11,12], multiple interactions [13], monoclonal antibodies with high affinity [14], etc., which enables efficient recognition of trace amounts of analytes. However, it typically trades off the regeneration ability of FETs as the toughly bonded analytes block the recognition sites and have low recovery efficiency compared to the unbound states [15]. To improve the regeneration ability, the interaction needs to weakened by using weak affinity interactions [16], decreasing the binding sites [17], exploiting an interlayer [18,19], etc., leading to relatively low sensitivity. Thus, the FET faces an inherent contradiction between sensitivity and regeneration ability. To the best of our knowledge, the limit of detection (LoD) is only at 10^−^^15^ mol L^−^^1^ level for regenerative FETs [20]. Considering the requirement in practical applications, it is highly desired to develop a strategy for designing FETs with both ultra-sensitivity and regeneration ability [21].

**Figure 1:**
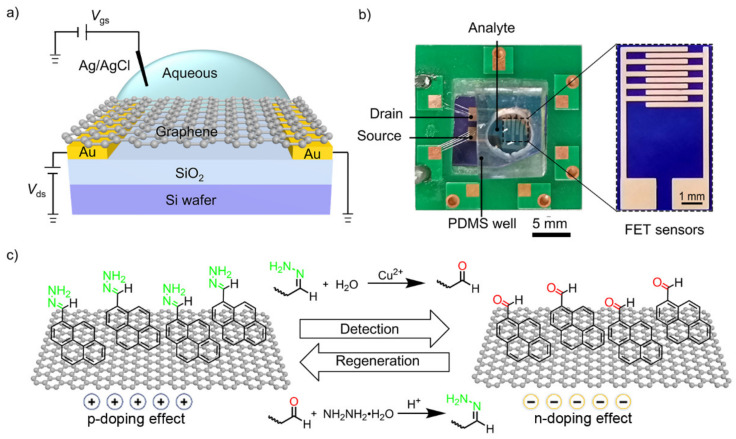
(**a**) Schematic structure of the PyCDH/graphene FET. (**b**) Image of the PyCDH/graphene FET and zoomed-in chip. The scale bar is 500 μm and 1 mm in length, respectively. (**c**) Proposed mechanism for PyCDH/graphene FET-based hydrazone bond.

Dynamic covalent chemistry (DCvC) is generally used in designing self-healing materials [22,23,24]. The DCvC concerns only reversible covalent bonds such as hydrazone bonds, boronate ester bonds, acylhydrazone bonds, and oxime bonds, which can be repeatedly broken and reformed, giving rise to the regeneration ability [25]. The DCvC usually requires the presence of catalysts to facilitate the reversible bond formation to reach a thermodynamic equilibrium in a reasonable time frame [26]. Herein, we develop an ultra-sensitive graphene FET with regeneration ability, which is enabled by reversible covalent reactions at the sensing interface (Figure 1). A dynamically reversible hydrolysis reaction of hydrazone bond catalyzed by copper (II) (Cu^2^^+^) is employed as a representative example [27,28]. After modifying the graphene surface with pyrene-1-carboxaldehyde hydrazone (PyCDH), Cu^2^^+^ efficiently hydrolyzes PyCDH to pyrene-1-carboxaldehyde (PyCHO), leading to an n-doping effect upon graphene (Figure 1c). The n-doping effect refers to channel conductivity changes induced by the charged analytes, which affects electric structures in graphene and generates a Donnan potential [2,7]. Consequently, the PyCDH/graphene FET detects Cu^2^^+^ with a LoD down to 5.0 × 10^−^^20^ mol L^−^^1^ even after 10 regenerative cycles. This is among the most sensitive regenerative FET sensors, showing the great potential of the DCvC-based sensing interface in future applications.

## 2. Materials and Methods

### 2.1. Materials and Characterization 

Copper foil (LOT: I26Z009) was purchased from Alfa Aesar a Johnson Matthey Co., Ltd. (Shanghai, China). 1-Pyrenecarboxaldehyde was purchased from Aladdin Chemistry Co., Ltd. (Shanghai, China). Hydrazine hydrate was purchased from Sinopharm Chemical Reagent Co., Ltd. (Beijing, China). Analytical grade metal salts were procured from Energy Chemical Co., Ltd. (Shanghai, China). The solution of PyCHO (5 mmol L^−1^) was prepared in DMSO/H_2_O (4:1 *v*/*v*). The ethanol solution of hydrazine hydrate (10 mmol L^−^^1^) was prepared by dissolving hydrazine hydrate (2 mL, 50% conc.) and HCl (100 μL, 37% conc.) in absolute ethanol. Mono-layer graphene was grown by thermal chemical vapor deposition on high-purity copper foils. After growth, graphene was transferred by a standard procedure using a PMMA temporary substrate by electrochemical delamination (bubbling transfer) method. The samples were measured by Raman (HORIBA XploRA, 532 nm layer), NMR (Bruker, 400 MHz, Brückner Textile Sales & Services (Shanghai) Co., Ltd., Shanghai, China), and laser scanning confocal microscope (Nikon, C2+, Nikon Instruments (Shanghai) Co., Ltd.).

### 2.2. Device Fabrication

The solution-gated graphene FET chemical sensor was fabricated on a SiO_2_ (285 nm)/Si wafer via a bilayer lift-off photolithography process, and a polydimethylsiloxane (PDMS) well containing the graphene FET chemical sensor was formed on the substrate to hold the analytical solutions [29]. First, the sacrificial layer (LOR3A) and photo-resistant layer (S1813) was spin-coated on the substrate using a spin coater, respectively. The parallel source and drain electrodes consisting of a Cr/Au structure (5 nm/40 nm) were defined on the SiO_2_/Si substrate using standard photolithography and metal deposition techniques. Then, the device was kept in Remover PG overnight at room temperature to remove the residual photoresist. The FET had a channel length of 100 μm and a width of 2 mm (W/L = 200). Next, the CVD graphene was transferred onto the substrate by the electrochemical delamination method. Finally, PyCDH was immobilized onto the graphene surface by π-π stacking to fabricate the PyCDH/graphene FET.

### 2.3. Electrical Measurement

The electrical signal was measured by a semiconductor parameter analyzer (Keysight, B1500A, Keysight Technologies (China) Co., Ltd., Beijing, China) and a probe station (Everbeing, PE-4, EverBeing Int’l Corp, Hsinchu, China) at room temperature. All the aqueous solutions were prepared using deionized water with a resistivity of 18.2 MΩ cm. The Ag/AgCl reference electrode was used as the liquid gate electrode. The gate voltage (*V*_g_) was set lower than ±400 mV (versus Ag/AgCl), and the *V*_ds_ was set to 25 mV to avoid any electrochemical reaction on the electrodes.

### 2.4. Regeneration

The graphene FET, which detected 5.0 × 10^−^^8^ mol L^−^^1^ Cu^2^^+^, was washed with deionized water three times after removing the resist solution. Then the ethanol solution of hydrazine hydrate (100 μL) was added to PDMS well to incubate the probe PyCDH at room temperature for 12 h. Finally, the FET was washed with deionized water three times and sealed with deionized water (100 μL) until the next use. The above-mentioned series of processes were regarded as one cycle.

## 3. Results and Discussion

To fabricate the PyCDH/graphene FET (Figure S1), monolayer graphene was transferred onto a SiO_2_/Si wafer with photo-lithographically patterned gold electrodes. The Raman spectrum of the graphene (Figure S2) had a G peak at ~1580 cm^−^^1^ and a 2D peak at ~2700 cm^−^^1^. The ratio of 2D/G was around 3.0 indicating the monolayer nature, and the absence of D peak was at 1350 cm^−^^1^ indicating the high quality of graphene [30]. Thereafter, PyCHO was functionalized onto the graphene surface, followed by condensation reactions with hydrazine hydrate to yield PyCDH that serves as the probe for Cu^2^^+^ recognitions.

**Figure 2:**
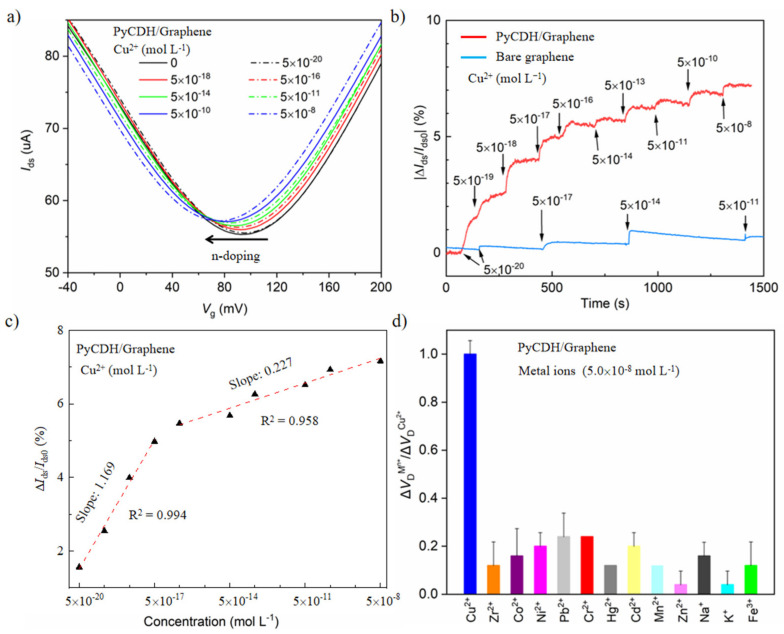
(**a**) Characteristic transfer curves (*V*_ds_ = 25 mV) of a PyCDH/graphene FET before and after the addition of Cu^2^^+^, when *V*_g_ varies from –40 to 200 mV. (**b**) Real-time *I*_ds_ response upon various concentrations of Cu^2^^+^ for the PyCDH/graphene FET (from 5.0 × 10^−^^20^ to 5.0 × 10^−^^8^ mol L^−^^1^, red line), and for bare graphene FET sensors (from 5.0 × 10^−^^20^ to 5.0 × 10^−^^11^ mol L^−1^, blue line). (**c**) The electrical responses versus the Cu^2^^+^ concentration for the PyCDH/graphene FET. (**d**) The VDirac shift change of the PyCDH/graphene FET in response to various metal ions: Cu^2^^+^, Zr^2^^+^, Co^2^^+^, Ni^2^^+^, Pb^2^^+^, Cr^2^^+^, Hg^2^^+^, Cd^2^^+^, Mn^2^^+^, Na^+^, K^+^, and Fe^3^^+^ (5.0 × 10^−^^8^ mol L^−^^1^).

To demonstrate the sensing performance, the transfer characteristics of the device were measured at a drain-source voltage (*V*_ds_) of 25 mV (Figure S3 and Figure 2a). The graphene FET showed a typical ambipolar character. The gate voltage (*V*_g_) at minimum drain-source current (*I*_ds_) corresponds to the Dirac point (*V*_Dirac_). The charge transfer between graphene and H_2_O leads to hole doping [31]; thus, the graphene shows a *p*-type behavior in the deionized water with a *V*_Dirac_ at 54 mV (Figure 1c). After being functionalized by PyCHO, the *V*_Driac_ negatively shifts by 18 mV; the *V*_Driac_ shifts positively by 4 mV after the formation of PyCDH. The different doping effect of PyCHO and PyCDH is caused by the electronic effect of the aldehyde group and hydrazone (C=NNH_2_). With increasing Cu^2^^+^ concentrations from 5.0 × 10^−^^18^ to 5.0 × 10^−^^8^ mol L^−^^1^, the *V*_Driac_ negatively shifted from 92 to 76 mV. Furthermore, the *I*_ds_ at *V*_Driac_ gradually increased from 55.97 to 57.18 μA (Figure 2a). The value of the *V*_Driac_ shift decreases with successively increasing Cu^2^^+^ concentration and gradually approaches a saturation value. It is probably attributed to the decreasing amount of PyCDH on the graphene surface.

The real-time response of the PyCDH/graphene FET to increasing Cu^2^^+^ concentrations is expressed as |(*I*_ds_-*I*_ds,0_)/*I*_ds,0_| vs. time, where *I*_ds,0_ is the drain current at *V*_g_ = 0 V (Figure 2b). The limit of detection reaches 5.0 × 10^−18^ mol L^−1^ Cu^2+^ in deionized water, 4–5 orders of magnitude lower than existing transistor sensors. Additionally, the device exhibits an average diagnosis time of 33 s. These results show that the PyCDH/graphene TEF offers excellent detection capability. In contrast, the bare graphene FET sensor shows negligible response upon Cu^2^^+^ with a concentration up to 5.0 × 10^−^^11^ mol L^−^^1^. The linear detection ranges are either a low concentration range from 5.0 × 10^−^^20^ to 5.0 × 10^−^^17^ mol L^−^^1^ and a high concentration range from 5.0 × 10^−^^16^ to 5.0 × 10^−^^8^ mol L^−^^1^, respectively (Figure 2c). The sensitivity obtained through a linear fitting for the low concentration range was five times as the high concentration range, which is probably associated with decreased PyCDH concentration leading to the hydrolysis reaction speed declining sharply on the channel surface. Compared with other sensors reported in the literature summarized in Table 1, the LoD of the graphene FET based on DCvC was roughly 4–5 orders of magnitude lower than the existing detection methods. As such, the device is susceptible to detecting Cu^2^^+^ in an aqueous solution.

To demonstrate the selectivity of PyCDH/graphene FET, 5.0 × 10^−^^8^ mol L^−^^1^ of metal ions (i.e., Zr^2^^+^, Co^2^^+^, Ni^2^^+^, Pb^2^^+^, Cr^2^^+^, Hg^2^^+^, Cd^2^^+^, Mn^2^^+^, Na^+^, K^+^, and Fe^3^^+^) were prepared. Moreover, the influence of ions on the transfer characteristics of the device was investigated with a normalized response (Figure 2d) when tested metal ions were added to deionized water, respectively. The PyCDH/graphene FET was found to give the highest *V*_Dirac_ shift caused by Cu^2^^+^ among the tested metal ions. The *V*_Dirac_ did not change clearly when Na^+^ and K^+^ were introduced into deionized water, while the Δ*V*_Dirac_ caused by Pb^2^^+^ was the highest among the interference ions. Interestingly, the Δ*V*_Dirac_ caused by Cu^2^^+^ was three times higher than that by Pb^2^^+^. On these grounds, the PyCDH/graphene FET confirmed the remarkable selectivity of Cu^2^^+^. The mechanism of the device to detect Cu^2^^+^ selectively is probably attributed to the efficient catalytic performance [40,41]. The response to Cu^2^^+^ rather than the anions presented in the solution was verified by measuring the response of FET on Cl^−^, NO^3^^−^, and SO_4_^2^^−^, where the shifts of *V*_Dirac_ remained constant (Figure S4). As such, the possible interference of anions can be excluded.

**Figure 3:**
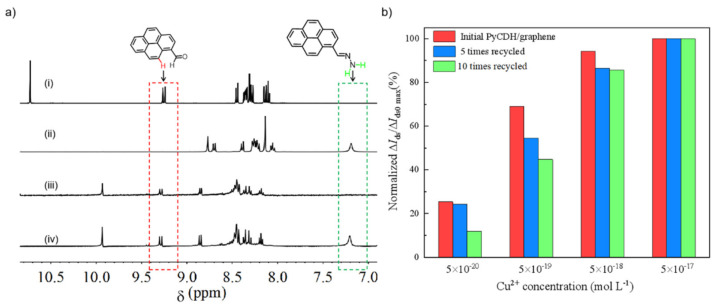
(**a**) ^1^H NMR spectra of PyCHO (i), PyCDH (ii), the product after Cu^2^^+^ detection (iii), and the product after regeneration (iv). (**b**) Regeneration ability of PyCDH/graphene FET.

To confirm that DCvC can reach regeneration as designed on the sensing interface, chemical and physical changes in the PyCDH probe were investigated. The transition from PyCHO to PyCDH was characterized via ^1^H NMR spectroscopy [22]. The C-10 proton peak of PyCHO at 9.29 ppm was quenched, while the amine proton of PyCDH at 7.21 ppm appeared (Figure 3a), suggesting the formation of PyCDH. When interacting with Cu^2^^+^, a signature doublet of PyCHO at 9.29 ppm occurred, and a broad singlet at 7.21 ppm disappeared, evidencing the reverse transition from PyCDH to PyCHO. The reversible DCvC was further investigated using a laser scanning confocal microscope (CLSM). The mean fluorescence intensity of graphene surface functionalized by PyCHO was about 635 arb.unit (a.u.), reaching 1023 a.u. after condensation reaction (Figure S5). When interacting with Cu^2^^+^, the mean fluorescence intensity declined to 512 a.u., which was similar to PyCHO. After regeneration by hydrazine hydrate, the mean fluorescence intensity heightened to 1042 a.u. That means the fluorescence phenomena of graphene FET finished a work cycle. The reversible CLSM results provide extra proof of the regeneration ability of the sensing interface. In particular, the regeneration ability of the sensing interface can be achieved by the presence of hydrazine hydrate to facilitate reversible bond formation. From the results observed, we propose a regenerative mechanism for the PyCDH/graphene FET shown in Figure 1c. The chemical probe PyCDH immobilized onto graphene surface by π-π stacking was hydrolyzed to PyCHO after achieving Cu^2^^+^ detection and then was regenerated by reacting with hydrazine hydrate to achieve a regenerative cycle.

To prove the mechanism we proposed, and to understand the role of DCvC, the regeneration ability of PyCDH/graphene FET was investigated at the low concentration range from 5.0 × 10^−^^20^ to 5.0 × 10^−^^17^ mol L^−^^1^ Cu^2^^+^ (Figure 3b). After 10 regenerative cycles, the sensitivity was roughly halved from 28% to 12% at 5.0 × 10^−^^20^ mol L^−^^1^. At the same time, the sensor remained an obvious real-time response of 18.0% for the whole concentration range from 5.0 × 10^−^^20^ to 5.0 × 10^−^^8^ mol L^−^^1^ Cu^2^^+^ (Figure S6). Therefore, employing DCvC to design FETs is an efficient strategy for fabricating an ultra-sensitive and regenerative device.

## 4. Conclusions

In conclusion, we report a regenerative testing methodology based on PyCDH/graphene FET sensors for the first time and address an urgent issue of ion testing that faces a trade-off between sensitivity and regeneration ability. Compared with other methods [33,34,36], the PyCDH/graphene FET sensors demonstrate reliable sensitivity and the impressive LoD (5.0 × 10^−^^20^ mol L^−^^1^) after 10 regeneration cycles. This technology achieves inspiring sensitivity, selectivity, and regeneration ability because reversible dynamic covalent bonds utilized in DCvC can be easily broken and reassembled to reform bonds. It might be the missing piece of the puzzle that enables ion sensors to be a comprehensive tool for not only sensitive testing but also regenerative applications in environmental monitoring and medical diagnoses.

## Figures and Tables

**Table 1:** Detection limit, technique, sensor material for analyte detection.

Analyte	Technique	Sensor Material	Detection Limit (mol L^−^^1^)	Recycle Times (Concentration, mol L^−^^1^)	Reference
Rh 6G	SERS	pMIP	1.0 × 10^−^^10^	8 times (1.0 × 10^−^^4^)	[32]
Cu^2^^+^	EC sensors	AuNPs-RBH	1.2 × 10^−^^14^	10 times (1.0 × 10^−^^8^)	[33]
Cu^2^^+^	Quantum dots	N-CQDs	1.0 × 10^−^^14^	Non-renewable	[34]
CaptAvidin	SWNT FET	TA-SWNT	5.0 × 10^−^^8^	8 times (1.4 × 10^−^^7^)	[35]
HVB DNA	NWs-FET	ssDNA	5.0 × 10^−^^15^	5 times (5.0 × 10^−^^11^)	[20]
Cu^2^^+^	Organic FET	Gly-Gly-His	1.0 × 10^−^^12^	Non-renewable	[36]
Procalcitonin	Organic FET	Anti-PCT	8.0 × 10^−^^13^	1 time (2.2 × 10^−^^12^)	[37]
Urea	Graphene FET	Urease-PEI	1.0 × 10^−^^6^	6 times (1.0 × 10^−^^4^)	[38]
Cu^2^^+^	Graphene FET	L-Phe	1.7 × 10^−^^13^	1 time (N.A.)	[39]
Cu^2^^+^	Graphene FET	PyCDH	5.0 × 10^−^^20^	10 times (5.0 × 10^−^^20^)	This work

Rh 6G, SERS, pMIP, EC, AuNPs-RBH, N-CQDs, TA-SWNT, HVB DNA, NWs, Anti-PCT, Urease-PEI, L-Phe, and N.A. indicated rhodamine 6G, surface-enhanced Raman scattering, porous molecularly imprinted polymer, electrochemical, gold nanoparticles modified with rhodamine B hydrazide, nitrogen-doped carbon quantum dots, tyrosine modified avidin with single-walled carbon nanotube, hepatitis B virus deoxyribonucleic acid, nanowires, anti-procalcitonin antibodies (monoclonal), Urease-polyethyleneimine, L-phenylalanine, and not available, respectively.

## Data Availability

Data is contained within the article or Supplementary Materials.

## Supplementary Materials

The following are available online at https://www.mdpi.com/article/10.3390/s22186947/s1: Figure S1 Schematic diagram of the device fabrication process [42,43], Figure S2. Raman spectrum of graphene, Figure S3. Characteristic transfer curves (Vds = 25 mV) of graphene FET sensor, Figure S4 Characteristic transfer curves of PyCDH/graphene FET sensor before and after different anions, Figure S5. Comparison of the mean fluorescence intensity (MFI) generated by graphene FET, Figure S6. Real-time Ids response upon various concentrations of Cu2^+^ ions for the regenerated PyCDH/graphene FET sensor.
