# Supplementary material for: Ultrasensitive and Regenerative Transistor Sensor Based on Dynamic Covalent Chemistry
Source: Sensors (Basel). 2022 Sep 14;22(18):6947. doi: 10.3390/s22186947 (PMC9505547; doi:10.3390/s22186947)
Supplement: Supplementary file 1 [file sensors-22-06947-s001.zip › sensors-1890841-supplementary.pdf]

Ultrasensitive and Regenerative Transistor Sensor Based on Dynamic Covalent Chemistry

Ban-Peng Cao [1,2,†], Changhao Dai [2,†], Xuejun Wang [2], Qiang Xiao [1] and Dacheng Wei [2,*]

[1] Jiangxi Key Laboratory of Organic Chemistry, Jiangxi Science and Technology Normal University, Nanchang 330013, China

[2] State Key Laboratory of Molecular Engineering of Polymers, Department of Macromolecular Science, Fudan University, Shanghai 200433, China

* Correspondence: weidc@fudan.edu.cn

† These authors contributed equally to this work.

Supporting Information (SI)

SUPPORTING FIGURES

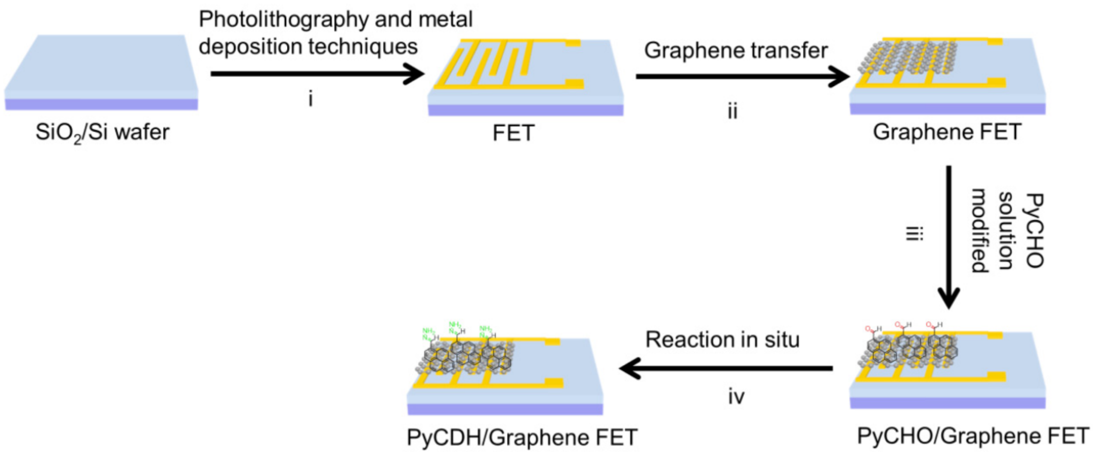

Figure S1. Schematic diagram of the device fabrication process. i) The LOR 3A and S1813 are spin-coated on the substrate at 4000 rpm with 1 min, respectively. After photolithography, the Cr/Au structure (5 nm/40 nm) is defined on the SiO_2/Si wafer to give FET. ii) Graphene produced by chemical vapor deposition is transferred to FET channel in 1 mol L^{-1} NaOH aqueous by electro-chemical delamination method [1,2]. iii) The graphene surface is modified by DMSO/H_2O (4:1) solution of PyCHO (5 mmol L^{-1}, 100 uL) in at room temperature with 2 hours. iv) PyCDH is synthesized in situ by ethanol solution of hydrazine hydrate (10 mmol L^{-1}, 100 uL) at room temperature with 12 hours to fabricate PyCDH/graphene FET.

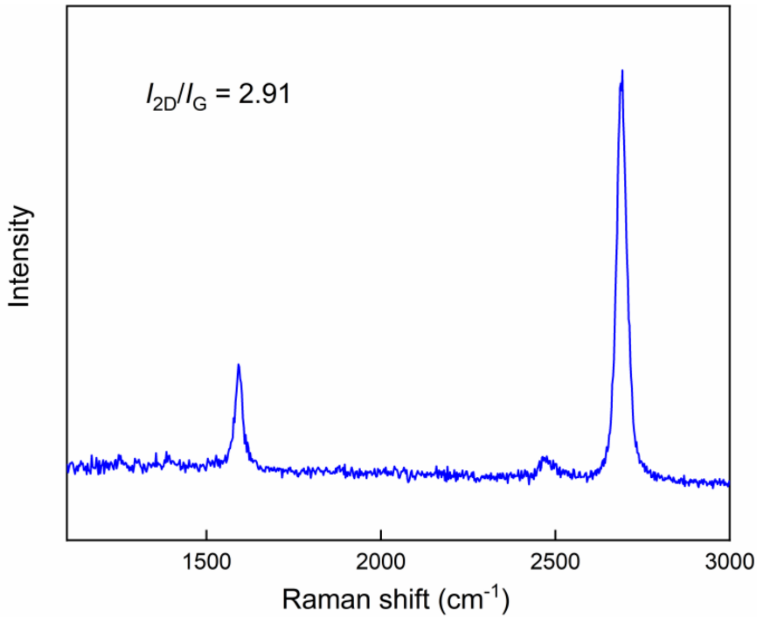

Figure S2. Raman spectrum of graphene

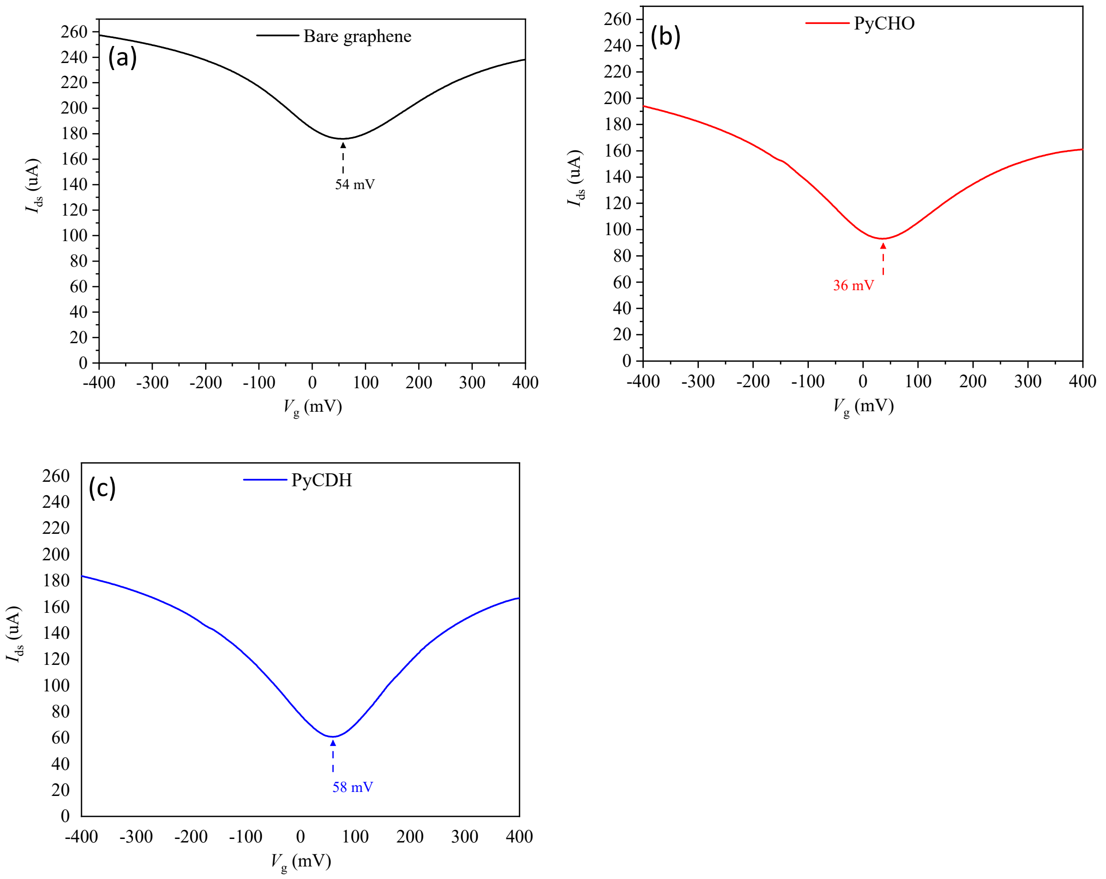

Figure S3. Characteristic transfer curves (V_{ds} = 25 mV) of graphene FET sensor (a) bare graphene, (b) modified by PyCHO, (c) modified by PyCDH

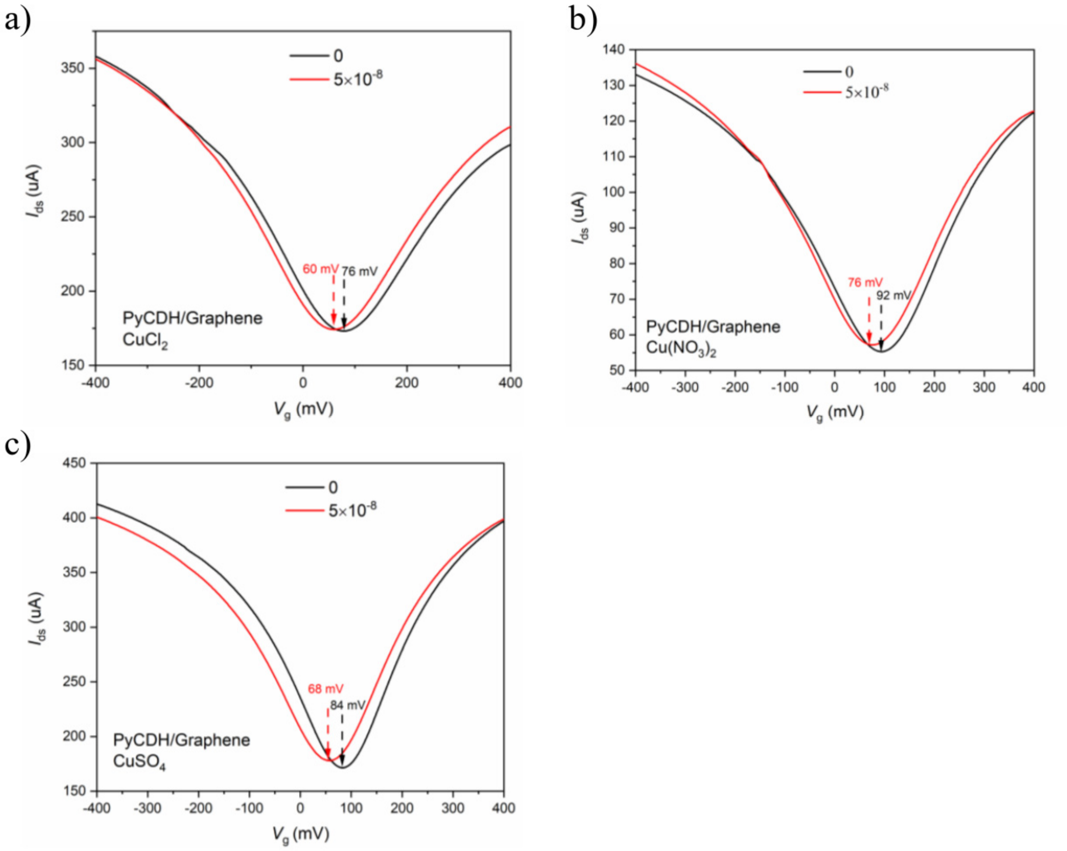

Figure S4. Characteristic transfer curves of PyCDH/graphene FET sensor before and after different anions (a) Cl^{-}, b) NO^{3-}, c) SO_4^{2-}) when Cu^{2+} at 5.0×10^{-8} mol L^{-1}

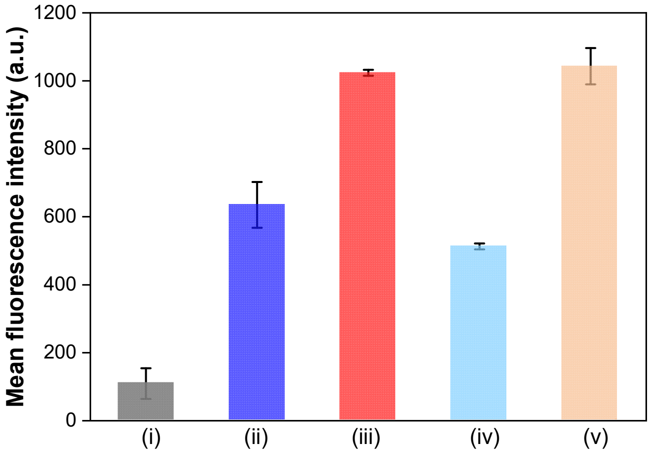

Figure S5. Comparison of the mean fluorescence intensity (MFI) generated by graphene FET. i) Graphene ii) Graphene surface modified by PyCHO. iii) Graphene surface modified by PyCDH. iv) PyCDH modified graphene FET after Cu^{2+} detected. v) Regenerated PyCDH modified graphene FET.

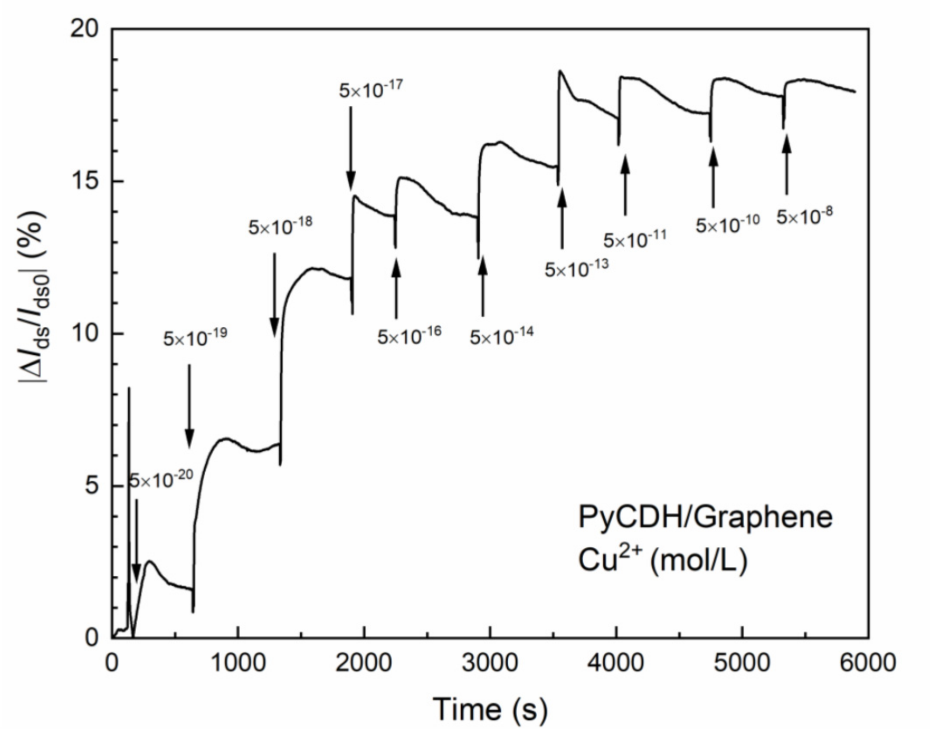

Figure S6. Real-time I_{ds} response upon various concentrations of Cu^{2+} ions for the regenerated PyCDH/graphene FET sensor (from 5.0×10^{-20} to 5.0×10^{-8} mol/L)

References

1. Yang, Y.; Yang, X.; Zou, X.; Wu, S.; Wan, D.; Cao, A.; Liao, L.; Yuan, Q.; Duan, X., Ultrafine graphene nanomesh with large on/off ratio for high-performance flexible biosensors. *Adv. Funct. Mater.* **2017,** *27* (19), 1604096.

2. De La Rosa, C. J. L.; Sun, J.; Lindvall, N.; Cole, M. T.; Nam, Y.; Löffler, M.; Olsson, E.; Teo, K. B. K.; Yurgens, A., Frame assisted H_2O electrolysis induced H_2 bubbling transfer of large area graphene grown by chemical vapor deposition on Cu. *Appl. Phys. Lett.* **2013,** *102* (2), 022101.
